# Design and evaluation of glutathione responsive glycosylated camptothecin nanosupramolecular prodrug

**DOI:** 10.1080/10717544.2021.1977424

**Published:** 2021-09-14

**Authors:** Wenhua Li, Zhong Chen, Xiaoying Liu, Mingming Lian, Haisheng Peng, Changmei Zhang

**Affiliations:** Department of Pharmaceutics, Daqing Campus of Harbin Medical University, Daqing, China

**Keywords:** Glutathione, camptothecin, glycosylated, nanosupramolecular prodrug, anti-tumor ability

## Abstract

A novel tumor-targeted glutathione responsive Glycosylated-Camptothecin nanosupramolecular prodrug (CPT-GL NSp) was designed and fabricated *via* a disulfide bond. The effects of glycoligand with different polarities on solubility, self-assembly, stability, cellular uptake, and glutathione responsive cleaving were explored, and an optimal glycosylated ligand was selected for nanosupramolecular prodrug. It has been found that CPT-GL NSp exhibited higher drug loading than traditional nanoparticles. Among of which maltose modified NSp had the strongest anti-tumor effects than that of glucose and maltotriose. CPT-SS-Maltose had a similar anti-tumor ability to Irinotecan (IR), but the superior performance in solubility, hemolysis, and uptake of HepG2 cells.

## 1. Introduction

Chemotherapy in cancer therapy still has poor bioavailability, rapid blood/kidney clearance, severe multidrug resistance, and many side effects on normal tissues and cells (Qin et al., 2017). Although researchers have built a variety of nano-drug delivery systems to overcome these limitations, traditional nanocarriers exhibit substantially poor drug loading capacity and great damage to normal tissues and cells due to their accumulation of the carrier materials during degradation and metabolism. To improve the delivery efficiency of drugs, researchers have developed a new amphiphilic small-molecule prodrug (ASMP) by directly combining anti-cancer drugs with small-molecule compounds (Cheetham et al., 2014; Ye et al., 2014; Hu et al., 2015; Jin et al., 2015; Ren et al., 2015; Takaoka et al., 2015; Tan et al., 2015; Wang et al., 2015, 2016; Lock et al., 2016; Zheng et al., 2016). ASMP self-assemble into nanosupramolecular anti-tumor prodrug through directional and reversible non-covalent interactions among the molecules. They can significantly enhance the solubility and stability of the drug and achieve higher drug loading (Yu et al., 2018). ASMP can also be decorated with corresponding ligands to achieve the feature of an active targeted delivery system (Huang et al., 2014). There is a great diversity of ASMP, but little attention has been paid to the influences of hydrophilic ligands with different polarities on solubility, stability, and characteristics of nanosupramolecular anti-tumor prodrug. Here, a novel tumor-targeted glutathione (GSH) responsive nanosupramolecular prodrug (NSp), namely glycosylated camptothecin nanosupramolecular prodrug (CPT-GL NSp), was fabricated by combining hydrophobic camptothecin (CPT) with hydrophilic targeted glycoligand *via* a disulfide bond. Based on the high expression of glucose transporter 1 (GLUT1) on tumor cytomembrane (Yamazaki et al., 2000), the effects of hydrophilic part within amphiphilic molecules on the self-assembly, glucose, maltose, and maltotriose molecules were selected to optimize the impacts of glycoside ligand on the solubility, self-assembly, stability, cellular uptake, intracellular cleave, and cell apoptosis of NSp.

## 2. Experimental section

### 2.1. Materials

Camptothecin (CPT), Irinotecan (IR), Glucose/Maltose/Maltotriose, Glutathone (GSH), Methyl Thiazolyl Tetrazolium (MTT), Annexin V-FITC, DRAQ5, DiO, Calcein AM were purchased from Innochem; Cell cycle kit was purchased from Beyotime Biotechnology; HepG2 cell line was donated by Peng’s Research. All materials or reagents used in this project are commercially available unless otherwise stated. Methanol and acetonitrile were chromatographic pure, and other reagents used in the synthesis experiment were commercial analytical or chemical purity. The reagents used in cell experiments were all commercial biochemical reagents, which were directly used after the purchase, and further purification operations were not carried out without special instructions.

Thin-Layer chromatography GF-254 (Merck, German) was used to monitor the reaction. UV light (254 or 365 nm) was used, and saturated iodine vapor was used as a general color reagent. The compounds were purified by column chromatography (200–300 mesh silica gel) and Sephadex LH-20. Nuclear magnetic resonance (NMR) was measured by the Analysis and Testing Center of Dalian University of Technology in the solvent of deuterium (CDCl_3_ or DMSO-d6). Transmission electron microscopy (TEM) (H7600 Hitachi, Japan) was performed by the Analysis and Testing Center of Harbin Medical University; UPLC-LCT Premier XE TOF (waters); HPLC, 1200 Series (Agilent); HPLC-ELSD (Shimadu); Freezer dryer [FD5-6, Jinximeng (Beijing) Instrument Co., Ltd.]; Nano-particle size and Zeta potential analyzer (Nano-ZS90, Malvern); Centrifuge (5430 R, Eppendorf); Rotary evaporators (EYELA); Automatic microplate reader (SYNERGY HTX, BioTek); Flow cytometry (SN AW29005, CytoFLEX S, Beckman Coulter); Delta Vision Ultra (C3655-157, General Electric Company).

### 2.2. Methods

#### 2.2.1. Synthesis of camptothecin-glycoligand

**Scheme 1.**
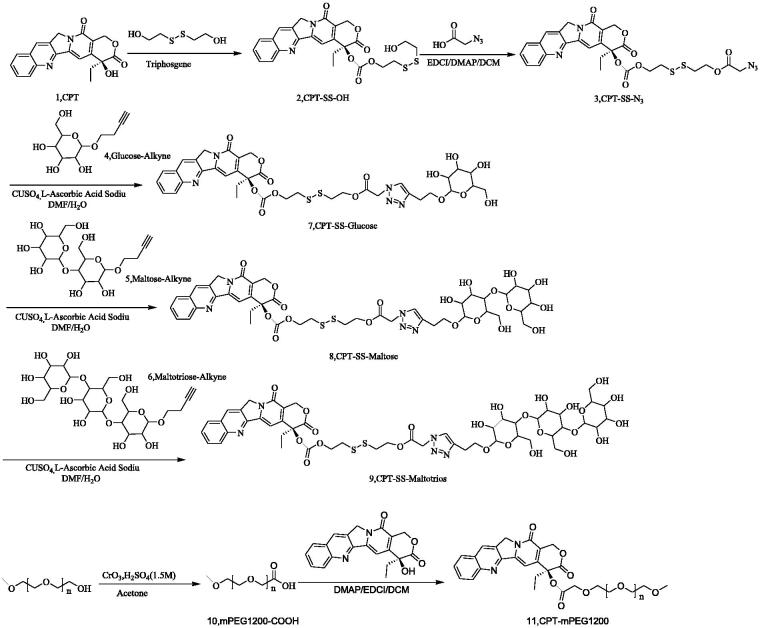
Synthesis of CPT-SS-Glucose/Maltose/Maltotriose and CPT-PEG1200.

The synthesis of CPT-PEG1200 starting from mPEG1200, the hydroxyl group of mPEG1200 was oxidized to a carboxyl group by chromium trioxide, then mPEG1200-COOH and CPT were connected by esterification reaction to afford CPT-PEG1200. The synthesis of CPT-SS-Glucose/Maltose/Maltotriose starting from CPT, CPT was linked to 2,2′-dithiodiethanol through triphosgene, then the azide group was introduced by esterification of CPT-SS-OH and azide acetic acid. Finally, CPT-SS-Glucose/Maltose/Maltotriose was obtained by clicking reaction with the intermediate compound (alkynyl-containing glucose, maltobiose, and maltotriose) (Scheme 1) (Cai et al., 2015). The reaction process and the observation results were monitored by TLC and UPLC-LCT. The purity of the final product was confirmed by HPLC-ELSD and ^1^H-NMR. The purification methods were mainly extraction, recrystallization, molecular sieve, column chromatography, and ion exchange.

##### 2.2.1.1. Cpt-SS-OH

Camptothecin (2 g, 5.75 mmol) and triphosgene (594 mg, 2 mmol) were dissolved in CH_2_Cl_2_ (150 mL) under an argon atmosphere in a three-neck flask protected from light. The mixture was stirred at room temperature for 10 min. A solution of DMAP (1.76 g, 2.5 mmol) and 2,2-dithiodiethanol (1.77 g, 11.5 mmol) in THF (10 mL) was added dropwise. The mixture was then stirred at room temperature overnight upon which time the solvent was removed by vacuum. The resulting crude mixture was dissolved in CH_2_Cl_2_ and then was washed with 1 mol/L aqueous HCl. The organic layer was dried over sodium sulfate, filtered, and removal of the excess solvent under reduced pressure to afford the pure product **2** (CPT-SS-OH) (2.7 g, 90%): ^1^H NMR (400 MHz, Chloroform-d): δ 8.43 (d, *J* = 12.2 Hz, 1H), 8.26 (d, *J* = 8.6 Hz, 1H), 7.98 (d, *J* = 8.3 Hz, 1H), 7.87 (td, *J* = 9.0, 8.5, 6.7 Hz, 1H), 7.71 (t, *J* = 7.7 Hz, 1H), 7.46 (s, 1H), 5.74 (d, *J* = 17.4 Hz, 1H), 5.42 (d, *J* = 17.3 Hz, 1H), 5.33 (d, *J* = 3.2 Hz, 2H), 4.50–4.33 (m, 2H), 3.94 (d, *J* = 7.1 Hz, 3H), 3.31 (s, 1H), 3.08–2.94 (m, 2H), 2.95–2.77 (m, 2H), 2.32 (dq, *J* = 15.0, 7.6 Hz, 1H), 2.19 (dq, *J* = 15.1, 8.0 Hz, 1H), 1.04 (t, *J* = 7.6 Hz, 3H). HRMS (ESI) Calcd for C_25_H_24_N_2_O_7_S_2_ [M + H]^+^ 529.1; found 529.1.

##### 2.2.1.2. Cpt-SS-N_3_

Compound **2** (1.7 g, 3.22 mmol), 2-Azidoacetic acid (163 mg, 1.61 mmol), EDCI (461 mg, 2.4 mmol), and DMAP (292.8 mg, 2.4 mmol) were dissolved in CH_2_Cl_2_ (30 mL) under an argon atmosphere in a round-bottomed flask protected from light. The mixture was stirred at room temperature overnight. The resulting crude mixture was washed with 1 mol/L aqueous HCl. The organic layer was dried over sodium sulfate, filtered, and removed the excess solvent under reduced pressure. The crude product was purified by flash column chromatography on silica gel (MeOH/CH_2_Cl_2_ 1:60) to afford the pure product **3** (CPT-SS-N_3_) (720 mg, 73%): ^1^H NMR (400 MHz, Chloroform-d): δ 8.43 (s, 1H), 8.25 (d, *J* = 8.5 Hz, 1H), 7.98 (d, *J* = 8.2 Hz, 1H), 7.87 (ddd, *J* = 8.4, 6.9, 1.5 Hz, 1H), 7.71 (t, *J* = 7.8 Hz, 1H), 7.36 (s, 1H), 5.73 (d, *J* = 17.2 Hz, 1H), 5.42 (d, *J* = 17.2 Hz, 1H), 5.33 (s, 2H), 4.60–4.30 (m, 4H), 3.87 (s, 2H), 2.96 (dt, *J* = 8.8, 6.7 Hz, 4H), 2.31 (dq, *J* = 14.8, 7.4 Hz, 1H), 2.18 (dq, *J* = 14.6, 7.4 Hz, 1H), 1.04 (t, *J* = 7.5 Hz, 3H). HRMS (ESI) Calcd for C_27_H_25_N_5_O_8_S_2_ [M + H]^+^ 612.1; found 612.1.

##### 2.2.1.3. Cpt-SS-Glucose

Compound **3** (100 mg, 0.164 mmol) was dissolved in DMF (5 mL) in a round-bottomed flask protected from light. A solution of compound **4** (38 mg, 0.164 mmol) in H_2_O (1 mL), 0.05 mol/L aqueous CuSO4 (100 μL, 0.05 mmol) and 0.05 mol/L aqueous sodium ascorbate (328 μL, 0.164 mmol) were added. The mixture was stirred at room temperature overnight. The solvent of the resulting crude mixture was removed by vacuum, dissolved in CH_2_Cl_2_, and then filtered out the insoluble impurities. The crude product was purified by molecular sieve chromatography on sephadex LH-20 (MeOH/CH_2_Cl_2_ 1:1) to afford the pure product **7** (CPT-SS-Glucose) (67 mg, 48.6%): ^1^H NMR (400 MHz, DMSO-d6): δ 8.72 (s, 1H), 8.17 (t, *J* = 9.7 Hz, 2H), 7.93 (s, 1H), 7.89 (t, *J* = 7.8 Hz, 1H), 7.74 (t, *J* = 7.5 Hz, 1H), 7.10 (s, 1H), 5.54 (d, *J* = 2.2 Hz, 2H), 5.32 (d, *J* = 9.6 Hz, 4H), 4.91 (d, *J* = 4.2 Hz, 1H), 4.73 (d, *J* = 4.9 Hz, 1H), 4.59 (t, *J* = 5.7 Hz, 1H), 4.43–4.27 (m, 5H), 4.15 (d, *J* = 6.8 Hz, 1H), 3.96 (q, *J* = 7.8 Hz, 1H), 3.68 (dd, *J* = 20.0, 10.8 Hz, 2H), 3.50 (tt, *J* = 11.1, 5.3 Hz, 2H), 3.29 (s, 2H), 3.01 (dt, *J* = 17.0, 6.3 Hz, 4H), 2.92 (t, *J* = 7.0 Hz, 2H), 2.19 (dd, *J* = 7.8, 3.3 Hz, 2H), 0.93 (t, *J* = 7.4 Hz, 3H). HRMS (ESI) Calcd for C_37_H_41_N_5_O_14_S_2_ [M + H]^+^ 844.2; found 844.2.

##### 2.2.1.4. Cpt-SS-Maltose

Compound **3** (100 mg, 0.164 mmol) was dissolved in DMF (5 mL) in a round-bottomed flask protected from light. A solution of compound 5 (65 mg, 0.164 mmol) in H_2_O (1 mL), 0.05 mol/L aqueous CuSO_4_ (100 μL, 0.05 mmol) and 0.05 mol/L aqueous Sodium ascorbate (328 μL, 0.164 mmol) were added. The mixture was stirred at room temperature overnight. The solvent of the resulting crude mixture was removed by vacuum, dissolved in CH_2_Cl_2_, and then filtered out the insoluble impurities. The crude product was purified by molecular sieve chromatography on sephadex LH-20 (MeOH/CH_2_Cl_2_ 1:1) to afford the pure product **8** (CPT-SS-Maltose) (80 mg, 48.6%): ^1^H NMR (400 MHz, DMSO-d6): δ 8.73 (s, 1H), 8.17 (t, *J* = 9.6 Hz, 2H), 7.93 (s, 1H), 7.89 (t, *J* = 7.8 Hz, 1H), 7.74 (t, *J* = 7.5 Hz, 1H), 7.11 (s, 1H), 5.54 (s, 3H), 5.47 (d, *J* = 6.2 Hz, 1H), 5.42–5.24 (m, 5H), 5.19 (d, *J* = 5.0 Hz, 1H), 5.02 (d, *J* = 3.7 Hz, 1H), 4.92 (t, *J* = 4.6 Hz, 3H), 4.53 (s, 2H), 4.44–4.22 (m, 6H), 3.99 (d, *J* = 8.6 Hz, 1H), 3.78–3.54 (m, 4H), 3.46 (t, *J* = 11.1 Hz, 1H), 3.00 (ddd, *J* = 34.4, 16.9, 7.9 Hz, 6H), 2.18 (s, 0H), 0.93 (t, *J* = 7.4 Hz, 3H). HRMS (ESI) Calcd for C_43_H_51_N_5_O_19_S_2_ [M + H]^+^ 1005.3; found 1005.3.

##### 2.2.1.5. Cpt-SS-Maltotriose

Compound **3** (100 mg, 0.164 mmol) was dissolved in DMF (5 mL) in a round-bottomed flask protected from light. A solution of compound 6 (91.2 mg, 0.164 mmol) in H_2_O (1 mL), 0.05 mol/L aqueous CuSO_4_ (100 μL, 0.05 mmol) and 0.05 mol/L aqueous Sodium ascorbate (328 μL, 0.164 mmol) were added. The mixture was stirred at room temperature overnight. The solvent of the resulting crude mixture was removed by vacuum, dissolved in CH_2_Cl_2_, and then filtered out the insoluble impurities. The crude product was purified by molecular sieve chromatography on sephadex LH-20 (MeOH/CH_2_Cl_2_ 1:1) to afford the pure product **9** (CPT-SS-Maltotriose) (110 mg, 57.6%): ^1^H NMR(400 MHz, DMSO-d6): δ 8.72 (s, 1H), 8.17 (t, *J* = 9.6 Hz, 2H), 7.93 (s, 1H), 7.89 (t, *J* = 7.7 Hz, 1H), 7.74 (t, *J* = 7.5 Hz, 1H), 7.11 (s, 1H), 5.54 (ddd, *J* = 18.2, 13.4, 6.9 Hz, 7H), 5.32 (d, *J* = 9.2 Hz, 4H), 5.22 (d, *J* = 4.9 Hz, 1H), 5.02 (dd, *J* = 12.4, 3.7 Hz, 2H), 4.91 (s, 2H), 4.55 (t, *J* = 9.9 Hz, 4H), 4.45–4.24 (m, 6H), 4.21–4.12 (m, 1H), 3.99 (q, *J* = 7.7 Hz, 1H), 3.85 (dd, *J* = 9.3, 4.2 Hz, 1H), 3.80–3.52 (m, 6H), 3.52–3.41 (m, 1H), 3.13–2.83 (m, 8H), 2.19 (dd, *J* = 7.7, 3.4 Hz, 1H), 0.93 (t, *J* = 7.4 Hz, 3H). HRMS (ESI) Calcd for C_49_H_61_N_5_O_24_S_2_ [M + 2H]^+^ 584.7; found 584.7.

##### 2.2.1.6. Mpeg1200-COOH

mPEG1200-OH (2.4 g, 2 mmol) was dissolved in acetone (20 mL) in a three-neck flask. A solution of CrO_3_ (600 mg, 6 mmol) in 1.5 mol/L aqueous H_2_SO_4_ (12 mL) was added dropwise. The mixture was stirred at room temperature overnight. The resulting crude mixture was dissolved in H_2_O, extracted with CH_2_Cl_2_, and concentrated under reduced pressure. The crude product was purified by ion-exchange chromatography on Amberlite^®^ IRA-410 (Cl) to afford the pure product **10** (mPEG1200-COOH) (1.58 g, 65.8%): ^1^H NMR (400 MHz, Chloroform-d): δ 4.17 (s, 2H), 3.67 (m, 3.85–3.50, 108H), 3.40 (s, 3H).

##### 2.2.1.7. Cpt-peg1200

Compound **10** (1.2 g, 1 mmol), DMAP (240 mg, 2 mmol), EDCI (384 mg, 2 mmol), and CPT (348 mg, 1 mmol) were dissolved in CH_2_Cl_2_ (20 mL) under an argon atmosphere in a round-bottomed flask protected from light. The mixture was stirred at room temperature for 3 days. The resulting crude mixture was washed with 1 mol/L aqueous HCl. The organic layer was dried over sodium sulfate, filtered, and removed the excess solvent under reduced pressure. The crude product was purified by flash column chromatography on silica gel (MeOH/CH_2_Cl_2_ 1:10) to afford the pure product **11** (CPT-PEG1200) (200 mg, 12.9%): ^1^H NMR (400 MHz, DMSO-d6) δ 8.72 (s, 1H), 8.24 (s, 1H), 8.23–8.09 (m, 1H), 7.89 (d, *J* = 7.8 Hz, 1H), 7.74 (d, *J* = 7.4 Hz, 1H), 7.12 (s, 1H), 5.52 (s, 1H), 5.32 (s, 1H), 4.19 (s, 6H), 4.14 (s, 3H), 3.65–3.60 (m, 8H), 3.55–3.46 (m, 252H), 3.45–3.41 (m, 8H), 3.24 (s, 8H), 1.24 (s, 4H), 0.93 (t, *J* = 7.1 Hz, 2H).

#### 2.2.2. Preparation of CPT-GL NSp

CPT-SS-Glucose, CPT-SS-Maltose, CPT-SS-Maltotriose, CPT-PEG1200 (1 mg/mL) were dissolved in deionized water. Every solution was diluted 100 times and ultrasonized under ice bath conditions (5/3/30/100 W). The mean particle size and zeta potential of each group were measured by a dynamic light scattering particle size analyzer. In addition, the potential changes of particle size were measured at 0, 2, 4, 6, 8, 12, 24, and 48 h, respectively to observe the stability. The prepared CPT-GL NSp were diluted with ultra-pure water and dropped on the copper grid, then stained with phosphotungstic acid (5%, w/v) and air-dried at room temperature. The morphology of CPT-GL NSp was characterized using TEM.

#### 2.2.3. Critical micelle concentration

The concentration gradients of CPT-SS-Glucose, CPT-SS-Maltose, CPT-SS-Maltotriose, CPT-PEG1200 were adjusted to 0, 5, 10, 20, 30, 40, 50, 60, 80, 90, 100, 120 (μM), and the solution volume was 4 mL. The surface tension of nanoparticles at different concentrations was measured by surface tensiometer (DCAT25, dataphysics), and the critical micelle concentration (CMC) was obtained by drawing the concentration-surface tension curve.

#### 2.2.4. Surface pressure

The surface pressure change of the nanoparticles was measured by using Langmuir-Blodgett (LB) film analyzer (KN2007, KSV NIMA, BiolinScientific). The nanoparticles (10 mM) were dissolved into trichloromethane and 30 μL solution was added into a pool of LB film analyzer. After drying the trichloride, the material formed a monolayer film on the water surface. Open the LB film analyzer to shrink the slider and extruded the monolayer film so as to obtain the compression area-water surface tension curve.

#### 2.2.5. Solubility

CPT was dissolved in DMSO to prepare a concentration gradient of 0, 5, 10, 20, 40, 60, 80, and 100 μg/mL. The UV absorption value at 360 nm was measured using a microplate analyzer, and the standard curve of CPT was drawn. CPT-GL NSp, CPT-PEG1200, and IR prodrug were dissolved in pure water. CPT was also dissolved in pure water but the removal of the undissolved components to obtain the saturated solution. The UV absorption value was detected at 360 nm, and the solubility was calculated according to the standard curve.

#### 2.2.6. Release property assay *in vitro*

Release study *in vitro* was performed in various concentrations of GSH (10, 1, and 0 mM). Typically, CPT-GL NSp was incubated with different concentrations of GSH for 1, 4, 8, 12, and 24 h. Then, the mixture was detected by HPLC and the cumulative release of CPT-GL NSp was analyzed by GraphPad Prism 8, respectively. CPT-GL NSp and CPT-PEG1200 were incubated with 10 mM GSH at 37 °C for 6 h. Then, particle size, zeta potential, and morphology of samples were measured (Chen et al., 2020).

#### 2.2.7. MTT assay

HepG2 cells were seeded at a density of 5.0 × 10^5^ cells/mL in 96-well plates and cultured for about 24 h. Then the culture medium in each well was replaced with 200 µL of DMEM, and drugs with different concentrations (0, 2, 5, 10, 20, 40, 60, 80, 100, and 200 μM) were added. After 24 h, 10 µL of MTT (5 mg/mL) was added to each well and the cells were incubated for another 4 h. Then, MTT-containing solution was removed and 100 µL of DMSO was added to dissolve the MTT formazan crystals. Finally, the absorbance at 490 nm of each well was recorded by an ELISA automatic microplate reader and the IC50 of each drug was calculated (Chen et al., 2020).

#### 2.2.8. Hemolytic test

Erythrocyte suspension (2%, 200 μL) were incubated with the different concentrations CPT-GL NSp, CPT-PEG1200, IR (0.5, 0.25, 0.125, 0.0625, and 0.03125 mg/mL) (200 μL) at 37 °C for 1 h. After centrifugal separation, the supernatant (100 μL) was collected and added to the 96-well plates, then was determined at 540 nm using UV–vis. The same volume of 200 μL distilled water and 200 μL normal saline was mixed with 200 μL 2% erythrocyte suspension as a positive control (100% hemolysis) and negative control (0% hemolysis), respectively (Zhu et al., 2021).

$$\text{Hemolytic}\;\text{rate}\;\%=(A_{\text{sample}}-A_{\text{negative}\;\text{control}})/(A_{\text{positive}\;\text{control}}-A_{\text{negative}\;\text{control}})\;\times \;100\%$$

#### 2.2.9. Cellular uptake analysis

The cellular uptake efficiency of drugs was studied in HepG2 cells. A density of 1.0 × 10^5^ cells/mL was cultured in 12-well plates for 24 h, then the CPT-GL NSP, CPT-PEG1200, and IR (1 mM) were added with 20 μL per well. After 4 h incubation, the cells were washed twice with PBS, then added 0.25% trypsin. After centrifugal separation, the single-cell suspension was collected and measured by flow cytometry with the DAPI channel (Ding et al., 2014).

#### 2.2.10. Annexin V-fluorescein isothiocyanate (FITC)/PI staining

HepG2 cells (1 × 10^6^ cells/mL) were seeded into 12-well plates and treated with CPT-GL NSp, CPT-PEG1200, and IR (1 mM, 20 μL) for 12 h. Then, cells were centrifuged at 1000 rpm for 5 min, and the supernatant was discarded. The cells were then transferred to a 1.5 mL tube containing 195 µL of binding buffer, then 5 µL of FITC-conjugated Annexin V and 10 µL of propidium iodide (PI) were added. The cells were gently mixed in a vortexer, protected from light, and incubated for 10–20 min at room temperature. After incubation, stained cells were measured by flow cytometry and apoptosis was quantitatively confirmed by analyzing the percentage of early apoptotic cells using Annexin-VFITC/PI double staining (Mou et al., 2016).

#### 2.2.11. Cell cycle analysis

The logarithmic growth phase HepG2 cells (1 × 10^6^ cells/mL) were seeded into 12-well plates and treated with CPT-GL NSp, CPT-PEG1200, and IR (1 mM, 10 μL) for 12 h. Then, cells were centrifuged at 800 rpm for 5 min, washed twice with cold PBS, and fixed with 75% precooled ethanol at 4 °C for 4 h. After that, the fixed cells were washed with PBS again and stained with PI (50 μg/mL, 400 μL) and RNase A (100 μg/mL, 100 μL) for 30 min at 4 °C in the dark. Finally, the stained cells were detected using a flow cytometer (Ma et al., 2018).

#### 2.2.12. Cellular uptake imaging by confocal microscopy

The logarithmic growth phase HepG2 cells (1 × 10^5^ cells/mL) were seeded into 8-well plates and incubated with CPT-GL NSp, CPT-PEG1200, and IR (1 mM, 20 μL) for 4 h. Then, the cells were washed twice with PBS and stained with DiO (cytomembrane) and DRAQ5 (nuclear staining) (5 μM) for 5 min. Finally, the stained cells were washed twice with PBS and analyzed by Delta Vision Ultra. The cell nucleus is red, the cytomembrane is green, and CPT is blue (Yu et al., 2018).

#### 2.2.13. Calcein AM-PI staining

Calcein AM and propidium iodide (PI) staining (viability and necrosis) was performed using a Calcein AM/PI kit. The logarithmic growth phase HepG2 cells (1 × 10^5^ cells/mL) were seeded in an 8-well plate and incubated with CPT-GL NSp, CPT-PEG1200, and IR (1 mM, 20 μL) for 12 h. After removing the culture medium and a gentle washing twice with PBS, the cells were stained with assay solution containing Calcein AM and PI (2 μL/mL Calcein-AM and 1 μL/mL PI) and incubated at 37 °C for 30 min. Digital images of viable (green fluorescence-excitation wavelength: 494 nm, emission wavelength: 517 nm) and dead (red fluorescence-excitation wavelength: 535 nm, emission wavelength: 617 nm) cells in selected areas were visualized using Delta Vision Ultra.

### 2.3. Data analysis

Statistical analysis was conducted with SPSS 22.0 (SPSS Inc., Chicago, IL, USA). Data are shown as the mean ± *SD*. Comparison between the two groups was analyzed using unpaired Student's *t*-tests and comparison among there or more groups was conducted using two-way analysis of variance (ANOVA). *p* < .05 was considered statistically significant.

## 3. Results and discussion

### 3.1. Synthesis of CPT-GL

Three kinds of CPT-GL, namely CPT-SS-Glucose, CPT-SS-Maltose, and CPT-SS-Maltotriose, were faint yellow solid, while CPT-PEG1200 was white oily semisolid. The ^1^H-NMR of CPT-SS-Glucose, CPT-SS-Maltose, CPT-SS-Maltotriose, and CPT-PEG1200 were shown in Figures S1–S4. CPT-PEG1200 without disulfide bond was synthesized as the control to compare the responsiveness of GSH with CPT-GL NSp with a disulfide bond. The synthesis process of CPT-GL NSp was beneficial to industrial production due to its advantages of the short route, high purity, high yield, and easy operation. Moreover, the preparation of CPT-GL NSp was a simple and effective procedure in which CPT-GL NSp were spontaneously organized to form stable well-ordered structures after being dispersed in water using an ultrasonic bath at 400 W for 10 min.

### 3.2. Preparation of CPT-GL NSp

**Figure 1.**
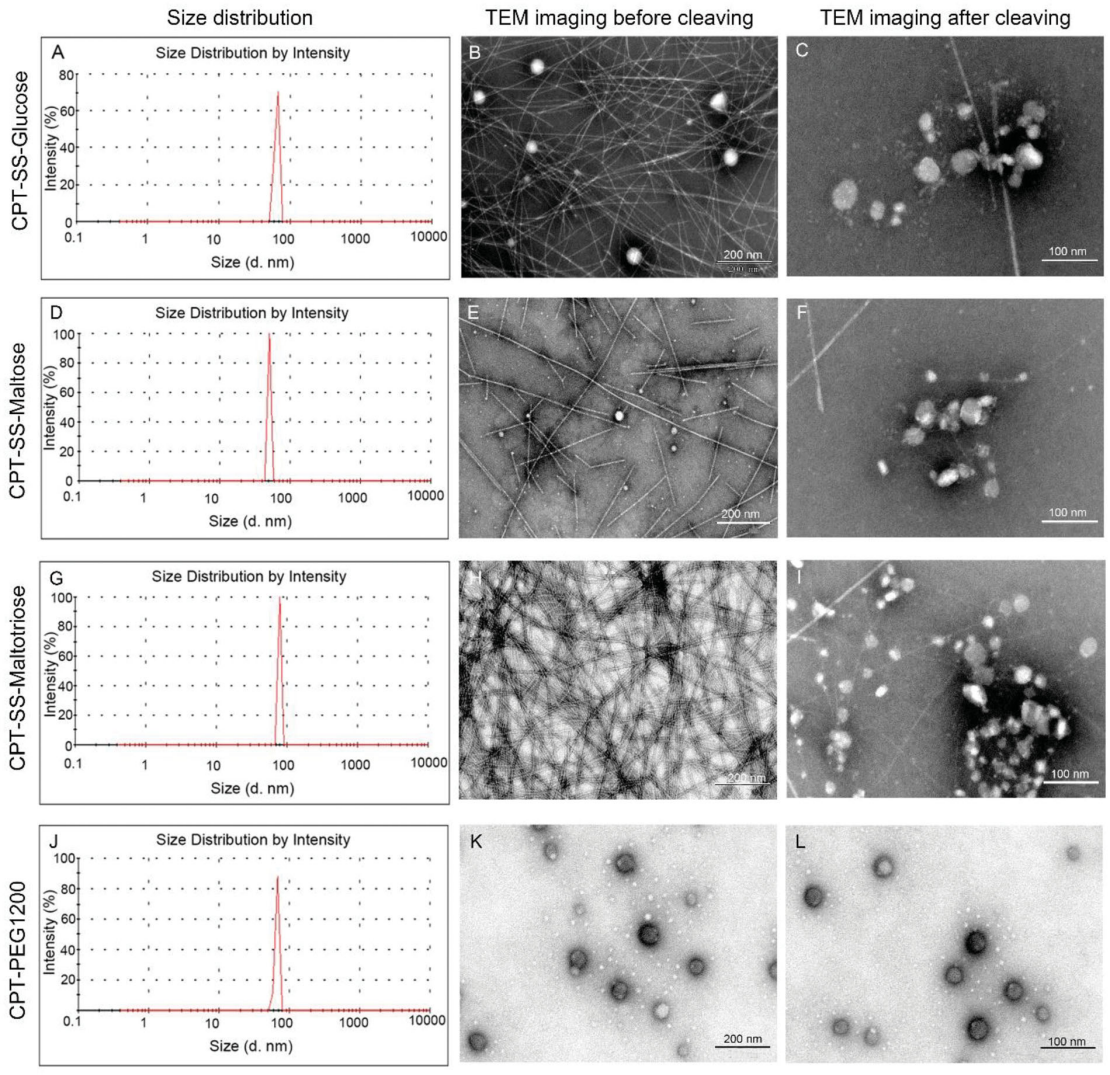
Size and morphology of CPT-GL NSp. (A,D,G,J) The particle sizes (d. nm); (B,E,H,K) The morphology of CPT-GL NSp before cleaving using a TEM; (C,F,I,L) The morphology of CPT-GL NSp using a TEM after being incubated with 10 mM GSH at 37 °C for 6 h.

The particle sizes of CPT-SS-Glucose, CPT-SS-Maltose, CPT-SS-Maltotriose, and CPT-PEG1200 were within 100 nm. Among them, CPT-SS-Maltose had excellent features in particle size, zeta potential, and polydispersity index (PDI) compared with those of CPT-SS-Glucose and CPT-SS-Maltotriose (Figures 1(A,D,G,J), Table 1). But there was no significant statistical difference. Transmission electron microscope (TEM) images revealed that CPT-GL NSp assembled into filamentous nanostructures in water. At high concentration, sometimes CPT-GL NSp was observed to be spherical nanoparticle (Figures 1(B,E,H)). However, the self-assembling CPT-PEG1200 was a spherical nanoparticle (Figure 1(K)). The disulfide bond of CPT-GL NSp was completely cleaved into CPT after being incubated with 10 mM GSH at 37 °C for 6 h. Therefore, filamentous nanostructures of CPT-GL NSp disappeared and be replaced with the aggregated CPT, demonstrating their excellent GSH responsiveness (Figures 1(C,F,I)). The morphology of TEM remained unchanged after incubation with GSH because CPT-PEG1200 had no disulfide bond (Figure 1(L)).

**Table 1.** The characteristics of various nanoparticles by size, ZP, PDI (*n* = 3 independent experiments).

Samples	Size (nm)	ZP (mV)	PDI
CPT-SS-glucose	65.28 ± 2.3	−24.4 ± 3.3	0.520 ± 0.32
CPT-SS-maltose	50.75 ± 4.7	−26.8 ± 2.7	0.370 ± 0.31
CPT-SS-maltotriose	78.82 ± 3.5	−22.4 ± 4.5	0.589 ± 0.36
CPT-PEG1200	66.99 ± 4.0	−26.2 ± 3.2	0.396 ± 0.23

### 3.3. Characteristic of CPT-GL NSp

**Figure 2.**
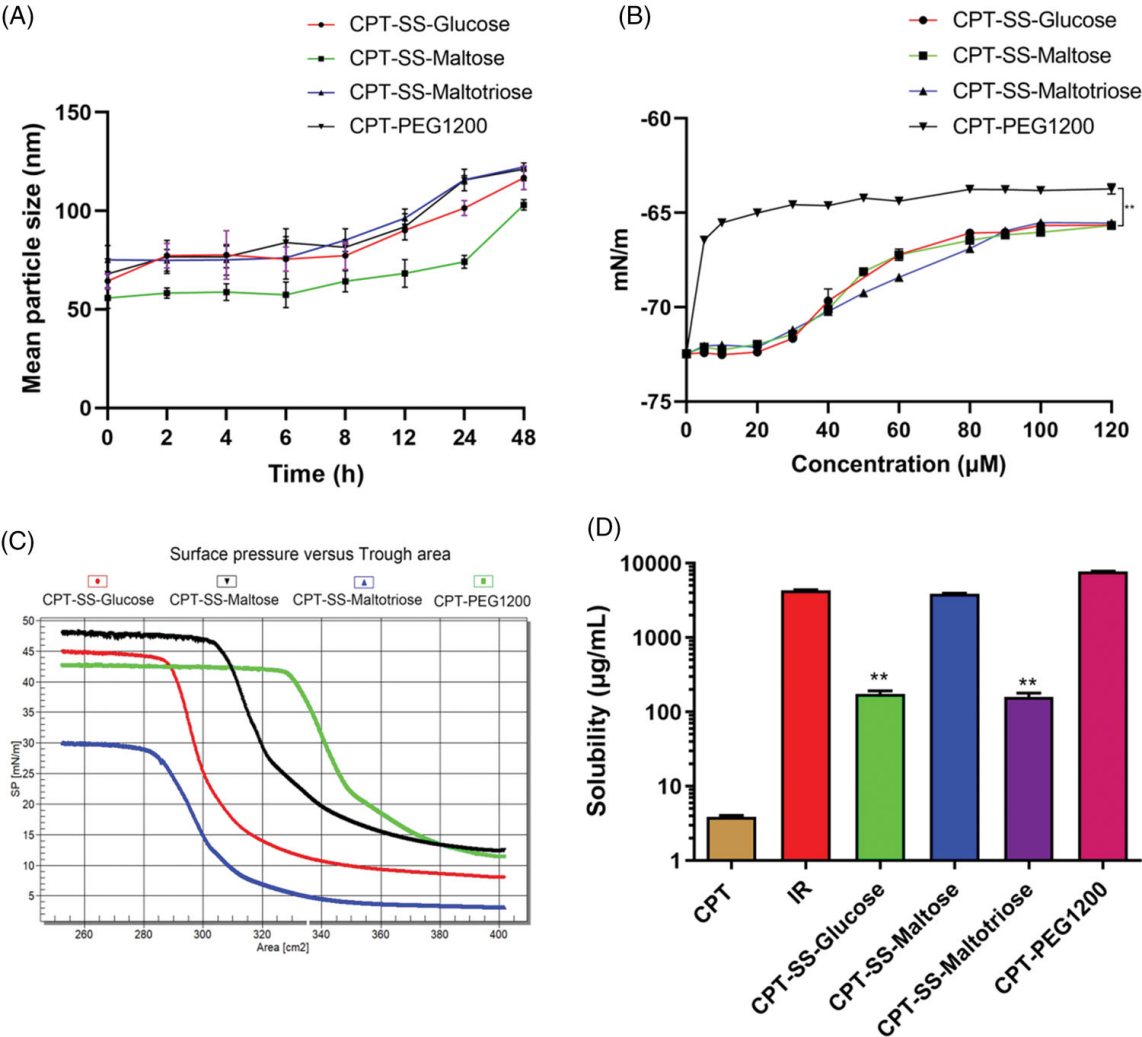
Characteristics of CPT-GL NSp. (A) Stability of different nanoparticles in 48 h; (B) Critical micelle concentration of different nanoparticles; (C) Change of surface pressure detected by Langmuir-Blodgett film analyzer; (D) Solubility of different nanoparticles (*n* = 3 independent experiments).

The average particle sizes of CPT-GL NSp and CPT-PEG1200 within 8 h were about 100 nm, which showed good stability (Figure 2(A)). Critical micelle concentrations (CMC) of CPT-SS-Glucose, CPT-SS-Maltose, and CPT-SS-Maltotriose were about 60, 60, and 90 μM, respectively, while that of CPT-PEG1200 was 10 μM (Figure 2(B)). Langmuir-Blodgett (LB) film analyzer was used to detect the changes of surface pressure, which showed that the trend of the curve of compression area-water surface tension was consistent with that of CMC (Figure 2(C)). CMC represents the ability of a material to assemble into micelles in water, which is consistent with their solubility. The smaller CMC has a much stronger self-assembly ability, resulting in greater solubility in water.

According to the standard curve (Figure S5), the solubilities of CPT-GL NSp, CPT-PEG1200, and IR prodrug were detected. We found that the solubility of CPT-SS-Maltose was similar to IR in water and obviously better than that of CPT-SS-Glucose and CPT-SS-Maltotriose (Figure 2(D)). CPT-GL NSp greatly increased the solubility of CPT. The best solubility of CPT-SS-Maltose may be due to the optimal ratio of the hydrophilic part and lipophilic part of ASMP. When the CPT part was the same, neither the oversize hydrophilic part (CPT-SS-Maltotriose) nor the oversize lipophilic part (CPT-SS-Glucose) was conducive to the solubility of ASMP. CPT-SS-Maltose had the best solubility because it had the best ratio of lipophilic and hydrophilic ends. The molecular weight of CPT is 348 Da while the molecular weight of the optimal sugar ligand, maltose, is 342 Da Comparatively, glucose (180 Da) and maltotriose (504 Da) are poor to form the nanoparticles, demonstrating the similar molecular weight units at both ends of AMSP are benefit to the self-assembly of nanoparticles and enhancing the solubility of CPT. hydrophile-lipophile balance (HLB) analysis based on the Langmuir-Blodgett membrane technology and solubility measurement protocol were applied to demonstrate the self-assembly structure of CPT-SS-Maltose. Additionally, as AMSP has the structure of surfactant molecules, HLB values of CPT-SS-Glucose, CPT-SS-Maltose, and CPT-SS-Maltotriose were estimated according to the Davies method to be 11.1, 16.5, and 21.9, respectively. Among them, the solubilization of CPT-SS-Maltose was the best which the HLB value was 16.5. Here, the length of 2,2′-dithiodiethanol used was moderate, and the bond angle/dihedral angle of 2,2′-dithiodiethanol was close to 90 degrees, which obviously increased the self-assembly ability and the stability of nanosupramolecular prodrug (Wang et al., 2014). The optimized ratio of the amphiphilic ends should not only increase the solubility of ASMP but also improve the stability of nanosupramolecular prodrug.

### 3.4. Cumulative release of CPT-GL NSp

**Figure 3.**
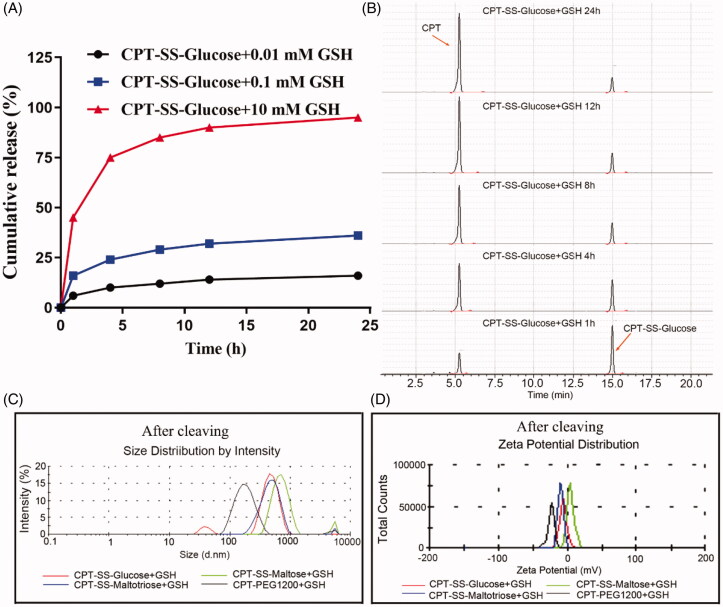
Characteristics of CPT-GL NSp after GSH response. (A) Cumulative release (%); (B) GSH sensitivity release in different times (GSH 10 mM); (C) Particle size after being cleaved by GSH (GSH 10 mM); (D) Zeta potential after being cleaved by GSH (GSH 10 mM) (*n* = 3 independent experiments).

Cumulative release due to the degradation of disulfide bond was highest when CPT-SS-Glucose was incubated with 10 mM GSH at 37 °C for 24 h (Figure 3(A)). CPT-SS-Maltose and CPT-SS-Maltotriose exhibited a similar trend (Figures S6, S7). The sensitivity of CPT-SS-Glucose incubated with 10 mM GSH was released at 1, 4, 8, 12, and 24 h. The peak area in the first hour was mainly CPT-SS-Glucose but gradually reduced with the increase of time, while the corresponding peak area of CPT gradually increases (Figure 3(B)). GSH sensitivity experiments proved that the disulfide bond of CPT-GL NSp was able to be cleaved by GSH in a concentration- and time-dependent manner. Appearances of CPT-SS-Glucose and CPT-SS-Maltotriose solution before cleaving were relatively cloudy. However, the colloidal solution of CPT-SS-Maltose and CPT-PEG1200 with better solubility were relatively clear and there was no precipitation after centrifugation at 10 000 g for 10 min. CPT-GL NSp was incubated with 10 mM GSH at 37 °C for 6 h, then some white precipitate was obtained after the same centrifugation (Figure S8). It indicates that the formation of free CPT due to that disulfide bond of CPT-GL NSp was cleaved. The particle size and zeta potential of CPT-GL NSp significantly became larger due to continuously particular aggregation after the oversaturation of free CPT (Figures 3(C,D)).

The content of overexpression GSH in tumor cells is much higher than that in normal cells (Yang et al., 2016). This feature enables the disulfide bond of CPT-GL NSp to be cleaved by GSH in the tumor, leading to the release of highly toxic CPT to kill tumor cells. The anti-tumor activity of CPT-GL NSp was improved and the systemic toxicity and long-term immunotoxicity were avoided (Zhang et al., 2020). In addition, CPT-PEG1200 showed no responsiveness of GSH due to without disulfide bond.

### 3.5. Cell assay evaluation

**Figure 4.**
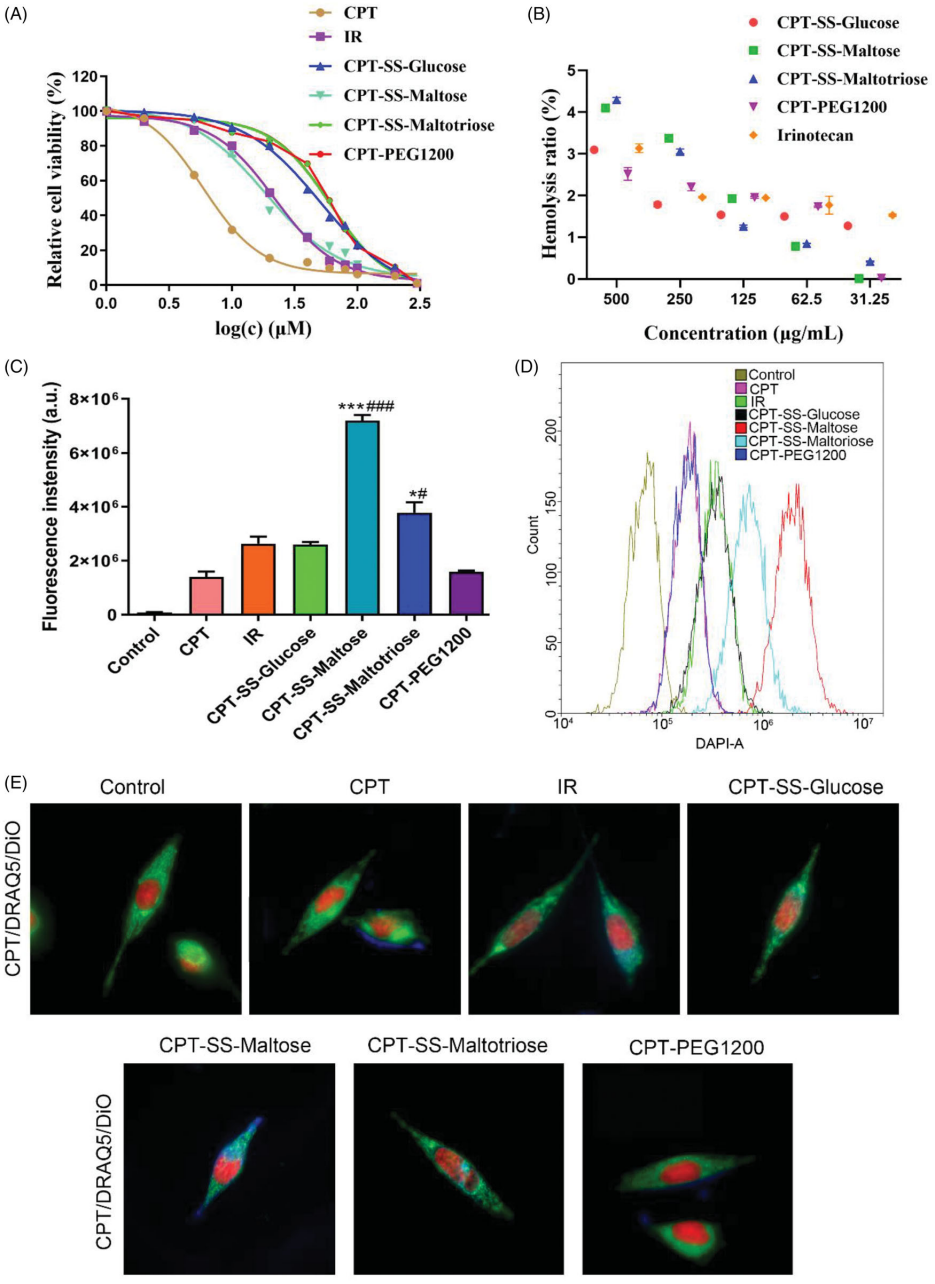
Cell experiments of CPT-GL NSp. (A) MTT assay; (B) Hemolysis ratio (%); (C,D) Cellular uptake by HepG2 cells using flow cytometry; (E) Confocal fluorescence images of CPT-GL NSp in HepG2 cells. The scale bar corresponding to 15 μm. CPT: blue; DRAQ5: red; DiO: green.

Cell assay evaluation of CPT-GL NSp showed that CPT dispersed in the DMSO had the strongest toxicity, but CPT could not be directly administered clinically because of its insoluble ability. IR has preferred water solubility and is currently the sole prodrug of CPT on the market. IR is a semisynthetic analog of camptothecin, originally isolated from the ornamental tree *Camptotheca acuminata*. IR was approved by the US Food and Drug Administration (FDA) in 1998 for the treatment of colon cancer. Although IR improves the water solubility and reduces the side effects of CPT, it is expensive and significantly reduces the anti-tumor ability of CPT (Xu and Villalona-Calero, 2002). Therefore, the research on effective delivery of CPT continues to focus on nano-prodrug. The cytotoxicity of CPT-SS-Maltose (IC50 = 18.47) was similar to IR (IC50 = 21.81) (Figure 4(A)), and CPT-GL NSp showed no hemolysis (Figure 4(B)). GLUT1 and asialoglycoprotein receptor (ASGPR) is overexpressed on the surface of tumor cells (Yamazaki et al., 2000; Li et al., 2008), such as HepG2 cells (D'Souza and Devarajan, 2015). Therefore, HepG2 cells were selected to research the cellular uptake capability of CPT-GL NSp. Flow cytometry demonstrated that more CPT-SS-Maltose was internalized by HepG2 cells (Figures 4(C,D)). Confocal images of HepG2 cells showed that CPT-SS-Maltose had the strongest signal of blue fluorescence of CPT (Figures 4(E), S9). This was because more CPT-SS-Maltose was taken by HepG2 cells and -SS- was cleaved into CPT after GSH response. The weakest blue fluorescence was CPT-PEG1200 without disulfide bond due to no glutathione-cleaved release of CPT.

**Figure 5.**
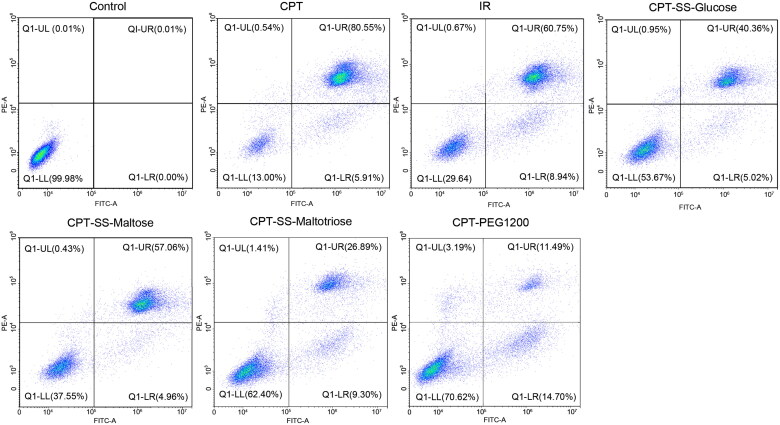
Flow cytometric analysis apoptosis of HepG2 cells with Camptothecin-Glycoligand nanosupramolecular prodrug (1 mM) after incubation for 12 h. Q1-LL: living cells; Q1-UR: late apoptotic cells; Q1-LR: early apoptotic cells.

**Figure 6.**
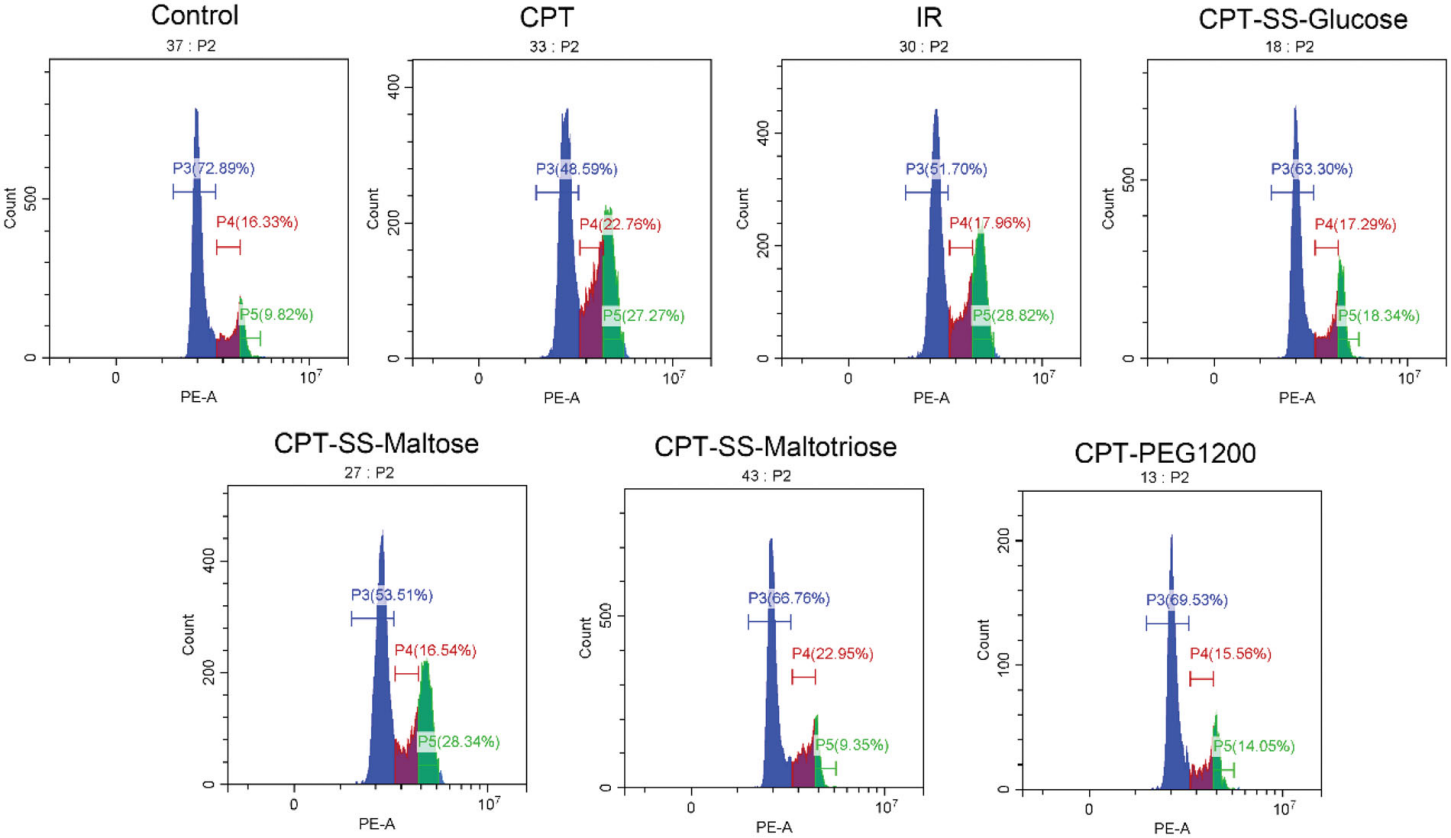
Effect of CPT-GL NSp on HepG2 cell cycle. Blue area is the G1-phase, purple area is the S-phase, green area is G2-phase.

**Figure 7.**
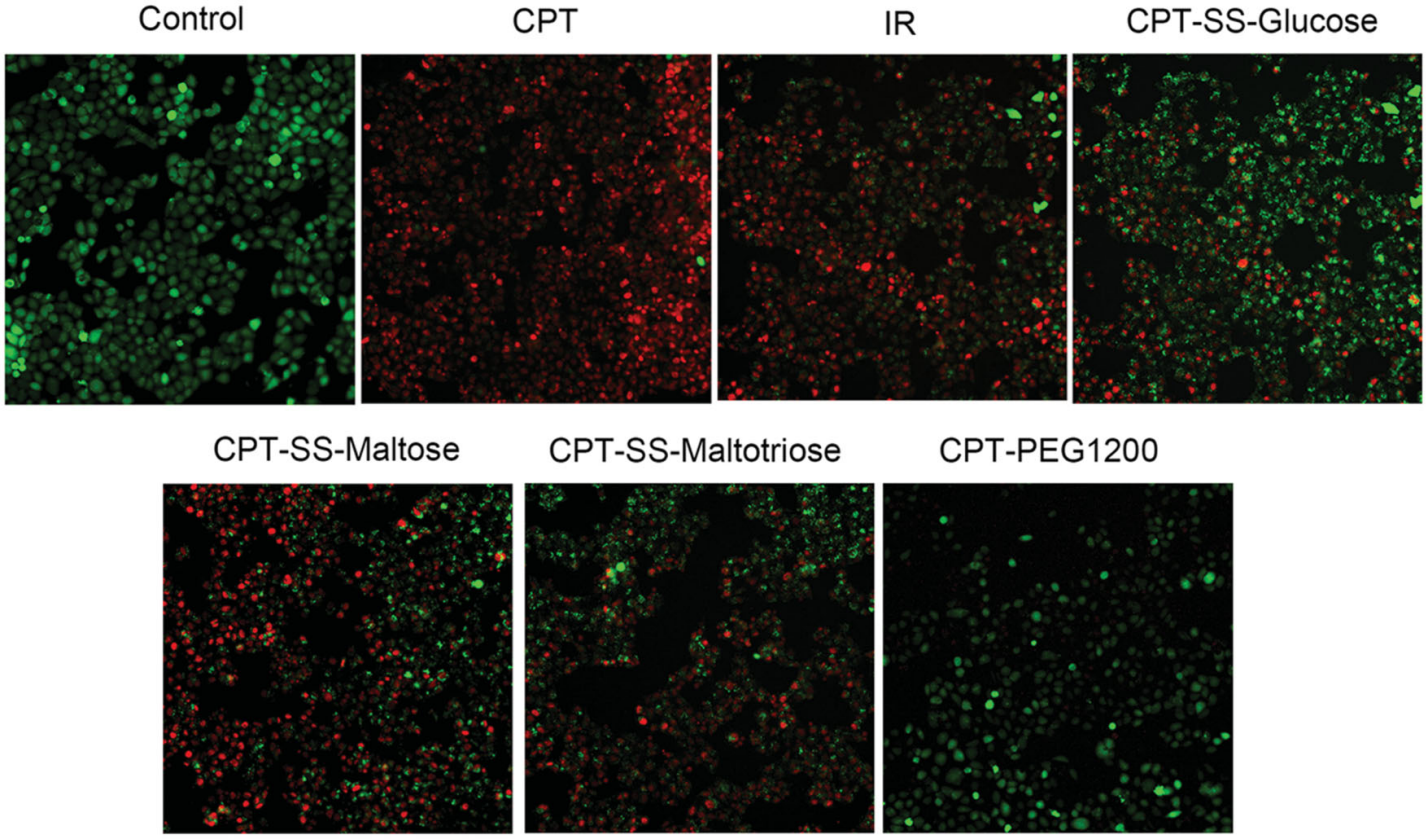
Fluorescence images of HepG2 cells cultured with CPT-GL NSp. Viable cells were stained with Calcein-AM (green), and dead/late apoptotic cells were stained with propidium iodide (PI) (red) (scale bar, 100 μm).

The evaluation strategy of drug cytotoxicity to tumor cells included the induced apoptosis of HepG2 cells, the inhibitory proliferation of HepG2 cells, and the judgment of live and dead cells stained by Calcein AM-PI dyes. The cell apoptosis rate of CPT-SS-Maltose was 55.24 ± 2.72%, which was similar to IR (62.50 ± 1.75%), indicating more ideal anti-tumor capability (Figures 5, S10, Table S1). Cell cycle experiments had confirmed that CPT-SS-Maltose had the same effects as IR, effectively blocking HepG2 cells in the S phase or G2 phase, inducing apoptosis, and inhibiting tumor cell proliferation (Figures 6, S11, Table S2). Calcein AM-PI staining revealed a large area of red fluorescence of cell death in the CPT group. CPT-SS-Glucose, CPT-SS-Maltose, and CPT-SS-Maltotriose were all able to induce cell death, which, however, the most potent was CPT-SS-Maltose. The cell death of CPT-PEG1200 was the least among all groups (Figures 7, S12). It showed that compared with the common CPT-PEG1200 self-assembling polymer-drug, CTP-GL NSp has better anti-tumor effects. Here, IR was used as a positive control. The data have verified that CPT-SS-Maltose has a similar anti-tumor ability to IR, but the superior performance in solubility, hemolysis, and uptake of HepG2 cells.

In a word, IR is the only CPT prodrug applied in the clinic, but it is not tumor-targeted and expensive. CPT-GL NSp not only have similar solubility in water with IR, but they are the targeted nano prodrug that has superior cellular uptake by HepG2 cells compared with IR. CPT-GL NSp exists in the form of a prodrug before reaching the tumor, with low toxicity, and can be passively and actively taken up by tumor cells after reaching tumor tissue, and then release highly toxic CPT after being cleaved by GSH. More importantly, the synthesis of CPT-GL NSp is very cheap and can be mass-produced.

## 4. Conclusion

In summary, the design of CPT-GL NSp was a confirmed strategy with higher drug loading than traditional nanoparticles. Among of which maltose modified NSp had the strongest anti-tumor effects than that of glucose and maltotriose. CPT-SS-Maltose has a similar anti-tumor ability to IR, but the superior performance in solubility, hemolysis, and uptake of HepG2 cells. Based on the Langmuir-Blodgett membrane technology and solubility measurement protocol, the superior self-assembly structural cause of CPT-SS-Maltose has been demonstrated, which provided new scientific ideas and the basis for the design of targeted nanosupramolecular anti-tumor prodrug.

## Supplementary Material

Supplemental Material
