# Supplementary material for: Design and evaluation of glutathione responsive glycosylated camptothecin nanosupramolecular prodrug
Source: Drug Deliv. 2021 Sep 14;28(1):1903–14. doi: 10.1080/10717544.2021.1977424 (PMC8462909; doi:10.1080/10717544.2021.1977424)
Supplement: Supplemental Material [file IDRD_A_1977424_SM8744.docx]

**Design and Evaluation of Glutathione Responsive Glycosylated Camptothecin Nanosupramolecular Prodrug**

Wenhua Li, Zhong Chen, Xiaoying, Liu, Mingming Lian, Haisheng Peng^*^ and Changmei Zhang^*^

Department of Pharmaceutics, Daqing Campus of Harbin Medical University, 1 Xinyang Rd, Daqing 163319, China

**3. Supplemental results**

**3.1 Synthesis of CPT-GL NSp**

Three kinds of GL-CPT, namely CPT-SS-Glucose, CPT-SS-Maltose, and CPT-SS-Maltotriose, were faint yellow solid, while CPT-PEG1200 was white oily semisolid. The ^1^H-NMR of CPT-SS-Glucose, CPT-SS-Maltose, CPT-SS-Maltotriose, and CPT-PEG1200 were shown in Figure S1-4


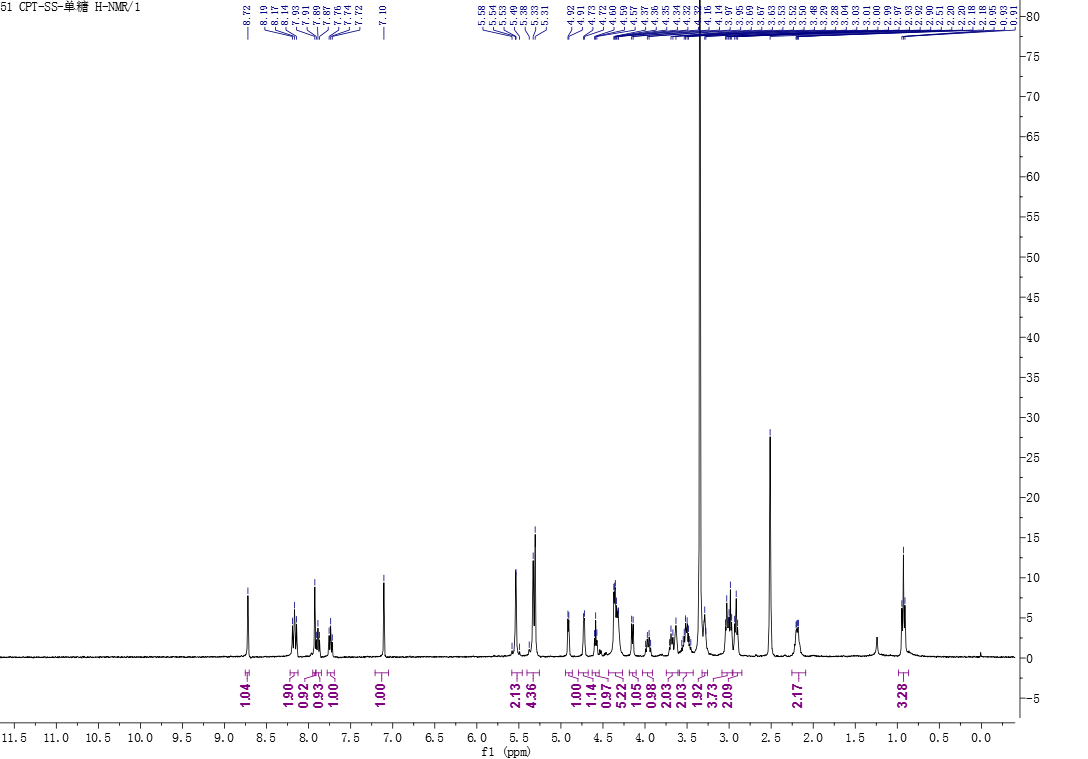

**Figure S1.**^1^H-NMR of CPT-SS-Glucose.


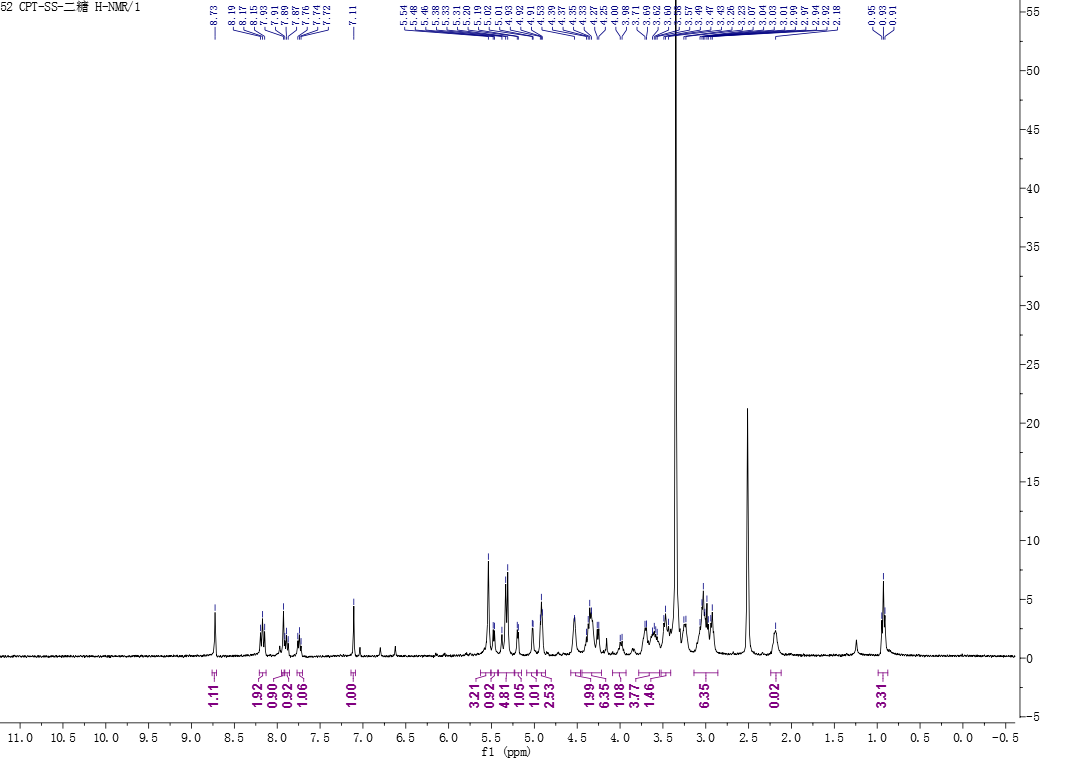

**Figure S2.** ^1^H-NMR of CPT-SS-Maltose.


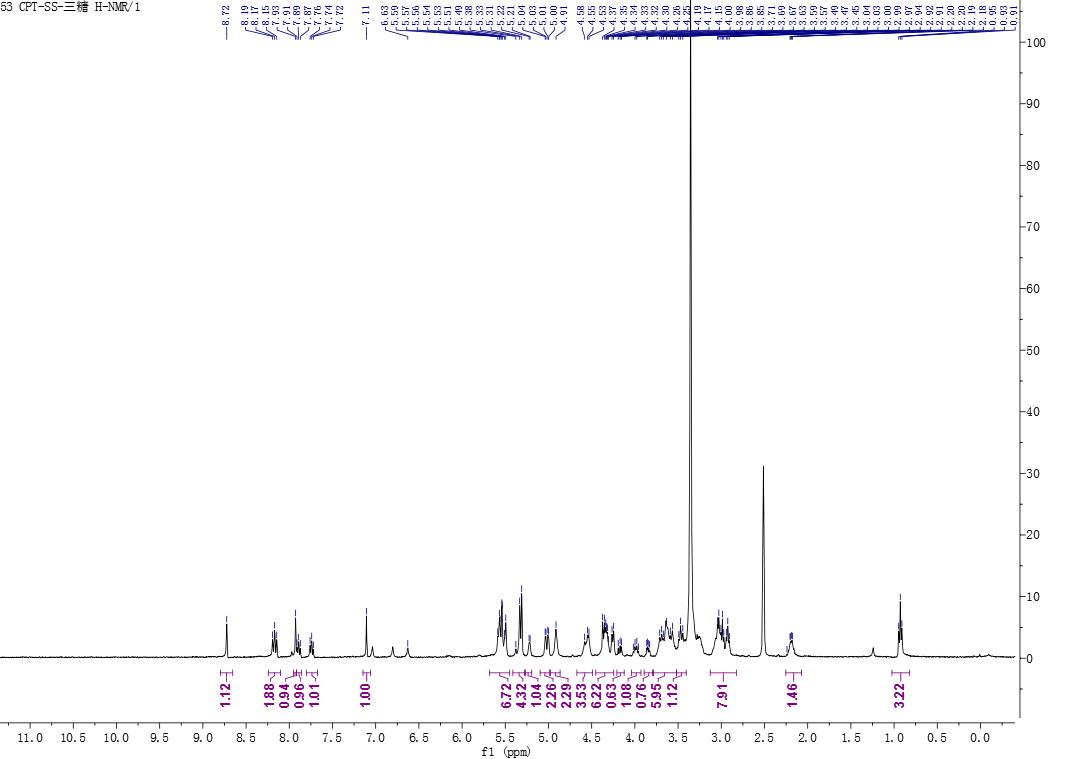

**Figure S3.** ^1^H-NMR of CPT-SS-Maltotriose.


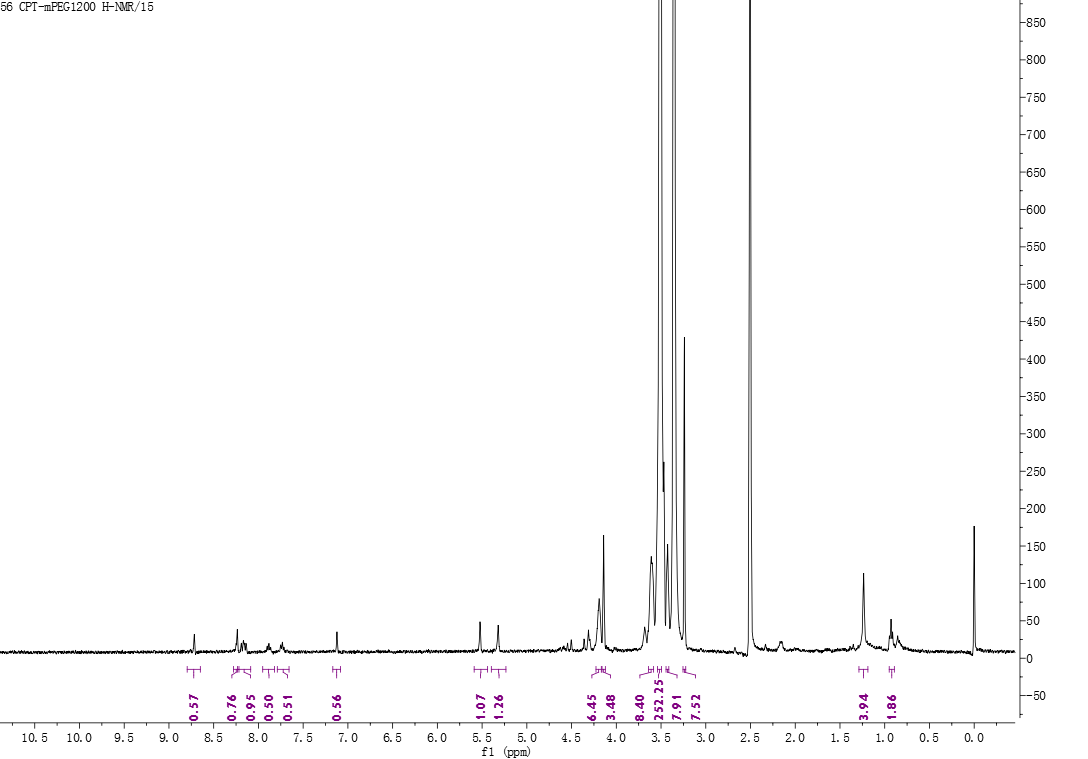

**Figure S4.** ^1^H-NMR of CPT-PEG1200.

**3.2 Characteristic of CPT-GL NSp**

**3.2.1 Solubility**

Solubilities of CPT-GL NSp, CPT-PEG1200 self-assembling polymer drug, IR prodrug were detected according to the standard curve of CPT which was shown in Figure S5.


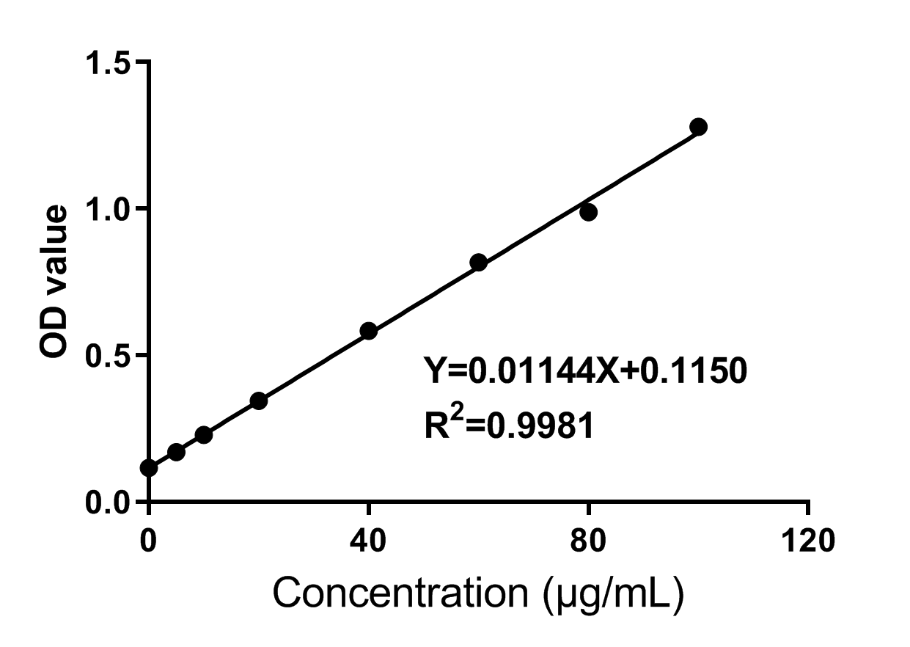


**Figure S5.** Standard curve for detection the solubility of nanoparticles.

**3.2.2 Cumulative release**

CPT-SS-Maltose and CPT-SS-[Maltotriose](C:/Program%20Files%20(x86)/Youdao/Dict/8.9.5.0/resultui/html/index.html#/javascript:;) were incubated with different concentrations of GSH (10, 1, 0.01 mM) for 24 h, then the cumulative releases were shown in Figure S6-7.


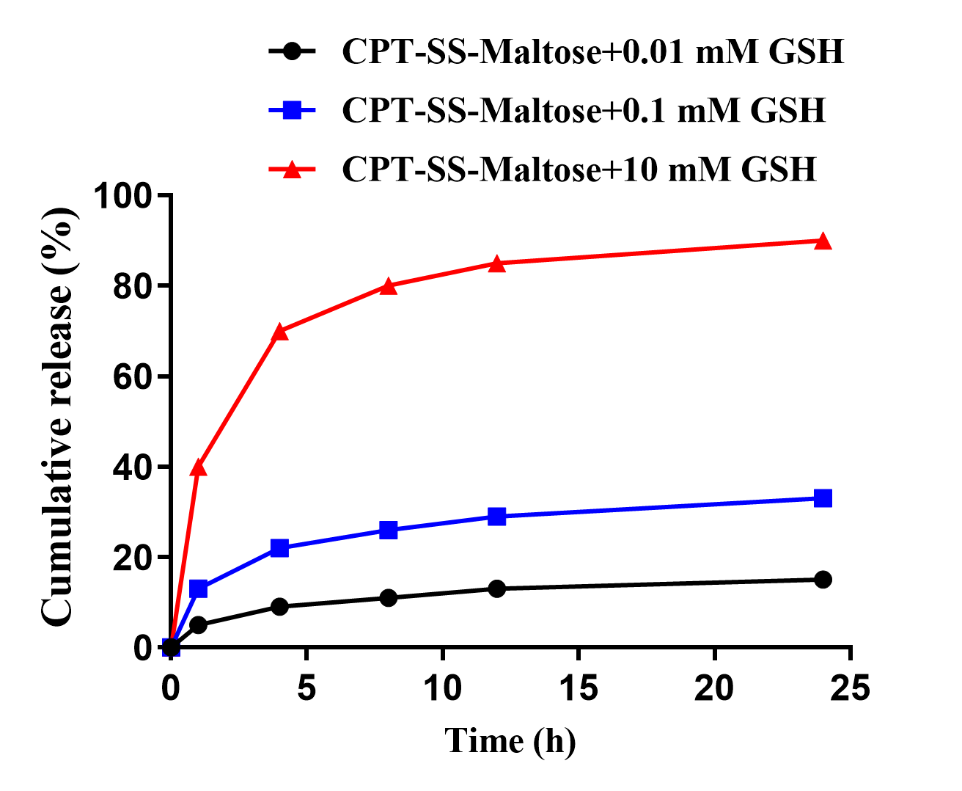


**Figure S6**. Cumulative release (%) of CPT-SS-Maltose with different concentrations of GSH.


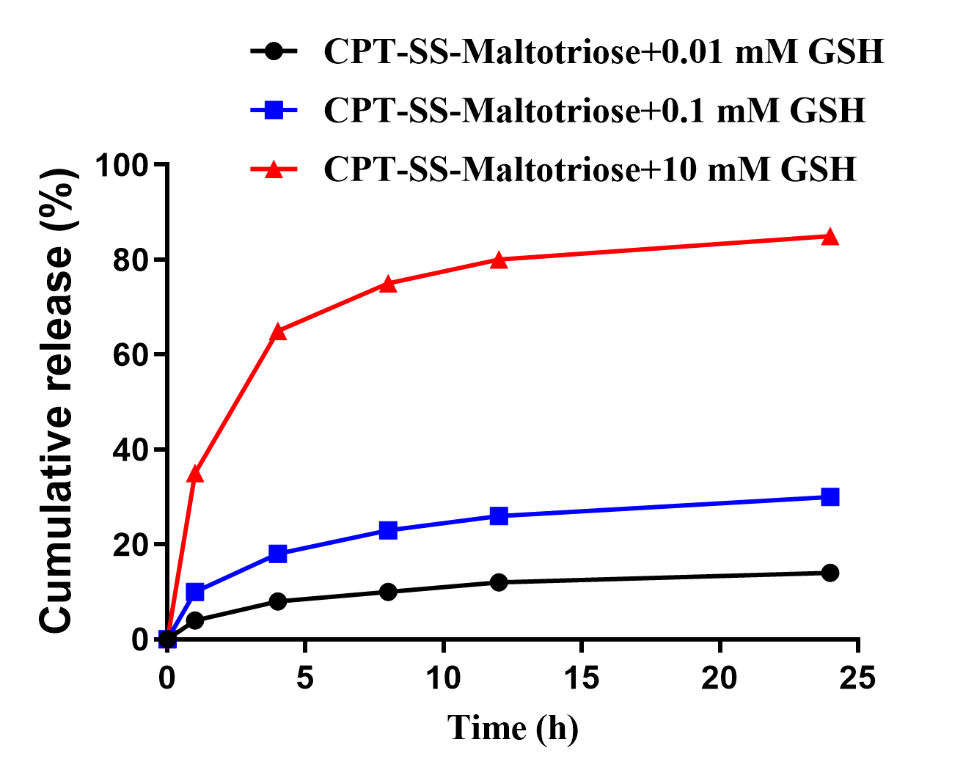


**Figure S7.** Cumulative release (%) of CPT-SS-Maltotriose with different concentrations of GSH.

CPT-SS-Glucose and CPT-SS-[Maltotriose](C:/Program%20Files%20(x86)/Youdao/Dict/8.9.5.0/resultui/html/index.html#/javascript:;) were muddy before cleaving, while CPT-SS-Maltose and CPT-PEG1200 had good solubility to be clear. No white precipitate occurred after centrifugation at 10000 rpm for 10 min. However, disulfide bond was cleaved after CPT-GL NSp incubating with 10 mM GSH at 37℃ for 6 h, and white precipitate appeared after centrifugation at 10000 rpm for 10 min, as shown in Figure S8.


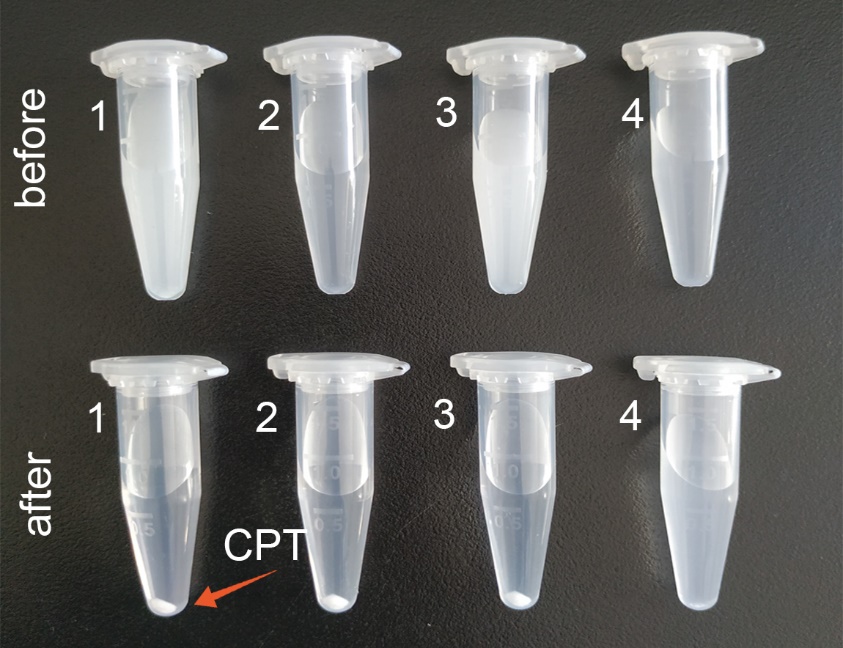


**Figure S8.** CPT-GL NSp were incubated with 10 mM GSH at 37℃ for 6 h, then centrifugated at 10000 rpm for 10 min to produce white precipitate. 1: CPT-SS-Glucose; 2: CPT-SS-Maltose; 3: CPT-SS-Maltotriose; 4: CPT-PEG1200.

**3.5** **Cell assay evaluation**

**1.** The column diagram of fluorescence intensity of Figure 4E is shown in Figure S9. Blue light indicates the fluorescence intensity of CPT, indicating cellular uptake of CTP.


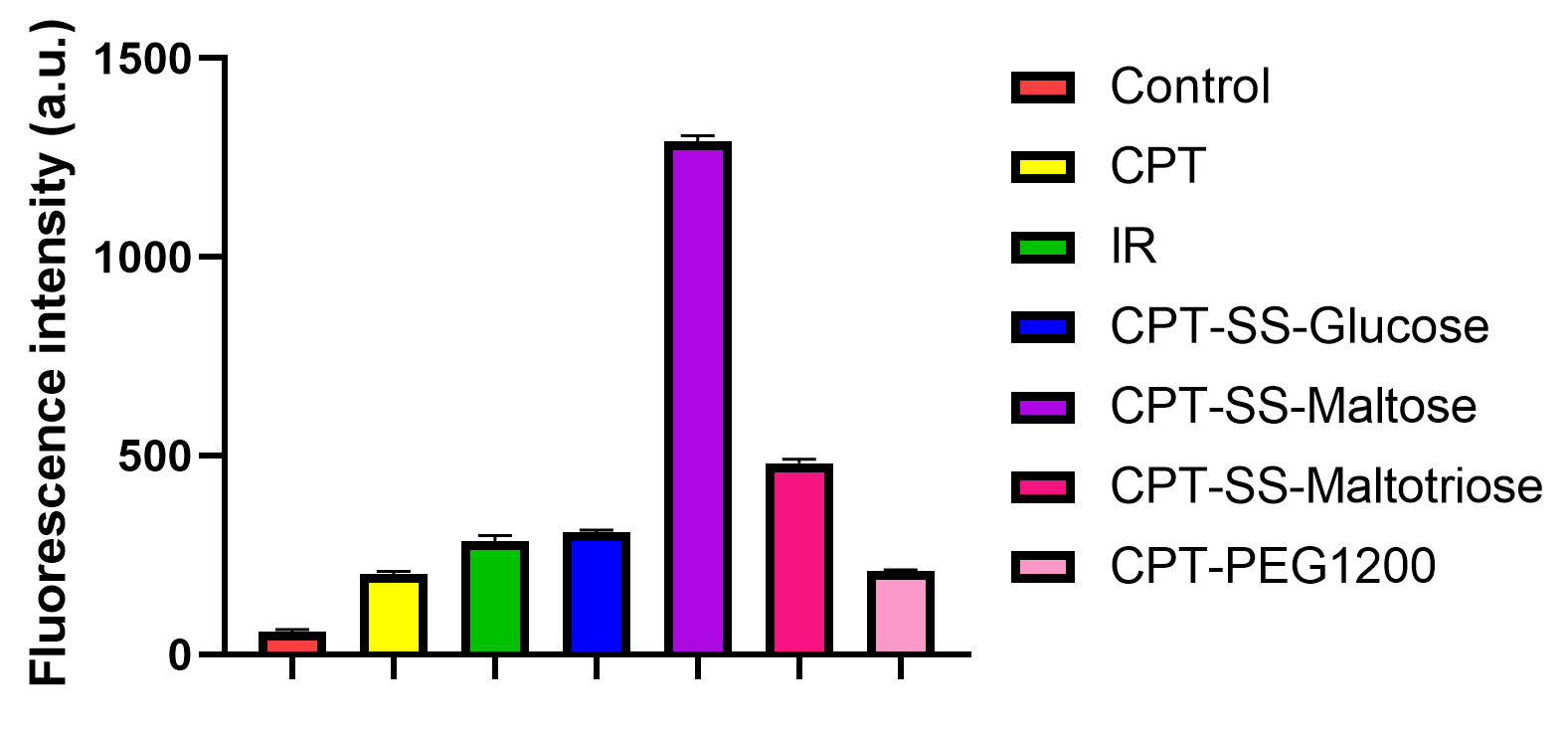


**Figure S9.** Fluorescence intensity of CPT, IR, and CPT-GL NSp.

**2.** The column diagram of fluorescence intensity of Figure 5 is shown in Figure S10, and specific value is shown in Table S1.


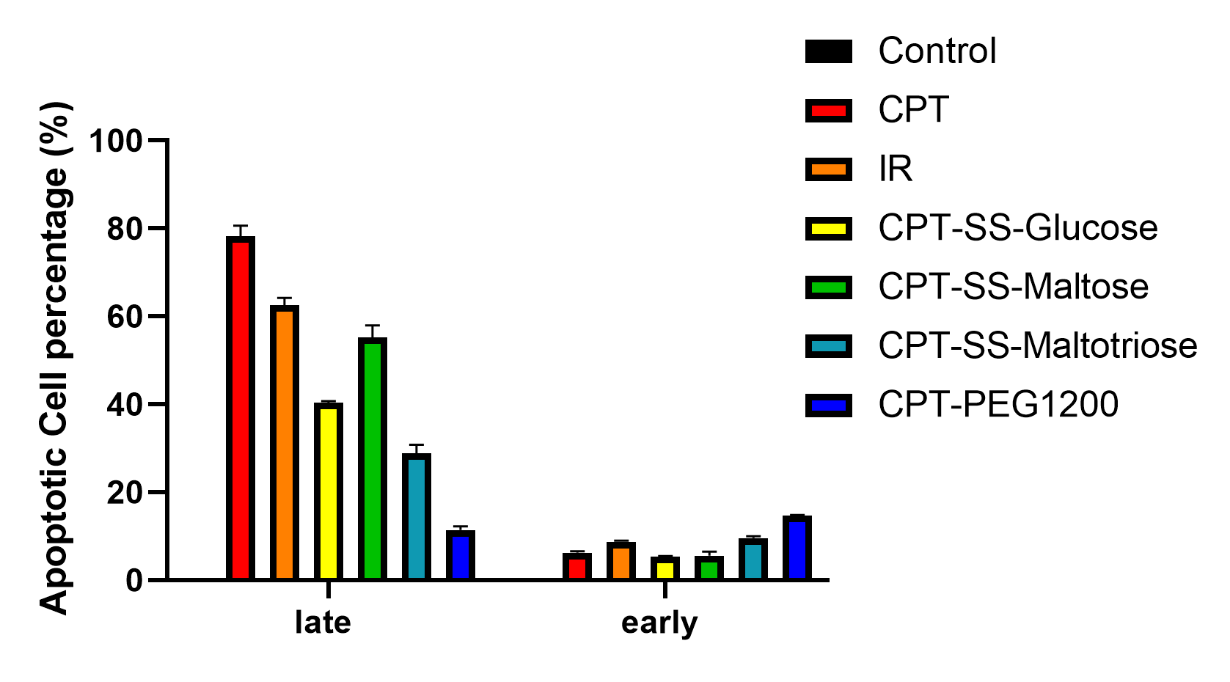


**Figure S10.** Late and early apoptotic cell percentage (%).

**Table S1.** Late and early apoptotic cell percentage (%).

| **Apoptotic cell percentage (%)** | **Control** | **CPT** | **IR** | **CPT-SS-Glucose** | **CPT-SS-Maltose** | **CPT-SS-Maltotriose** | **CPT-PEG1200** |
| --- | --- | --- | --- | --- | --- | --- | --- |
| Late | 0.00 | 78.19 ±2.42 | 62.5±1.75 | 40.39±0.28 | 55.24±2.72 | 28.76±2.06 | 11.40±0.80 |
| Early | 0.00 | 6.24±0.34 | 8.74±0.25 | 5.25±0.24 | 5.53±0.89 | 9.56±0.42 | 14.74±0.04 |

**3.** The column diagram of fluorescence intensity of Figure 6 is shown in Figure S11, and specific value is shown in Table S2.


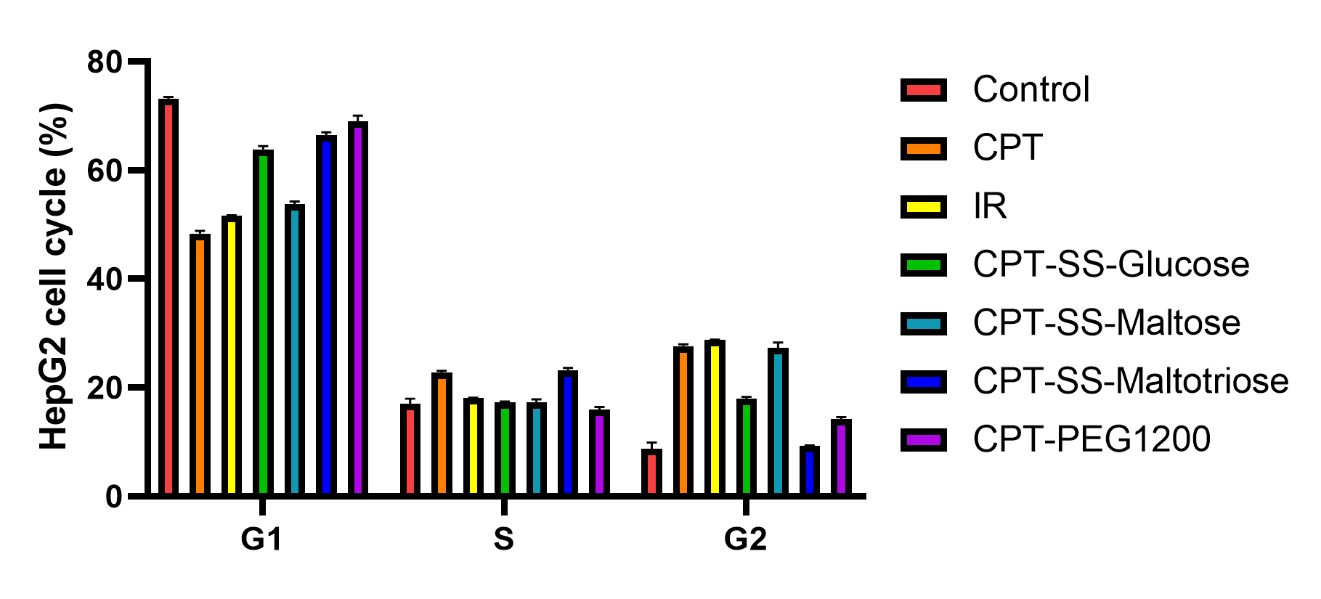


**Figure S11.** Effect of CPT, IR, and CPT-GL NSp on HepG2 cell cycle.

**Table S2.** Effect of CPT, IR, and CPT-GL NSp on HepG2 cell cycle (%).

|  | **Control** | **CPT** | **IR** | **CPT-SS-Glucose** | **CPT-SS-Maltose** | **CPT-SS-Maltotriose** | **CPT-PEG1200** |
| --- | --- | --- | --- | --- | --- | --- | --- |
| G1 | 73.19±0.29 | 48.3 ±0.56 | 51.55±0.13 | 63.86±0.59 | 53.76±0.46 | 66.45±0.53 | 68.98±1.03 |
| S | 17.03±0.94 | 22.75±0.37 | 18.00±0.19 | 17.27±0.18 | 17.23±0.61 | 23.21±0.42 | 15.98±0.44 |
| G2/M | 8.78±1.09 | 27.61±0.34 | 28.75±0.10 | 17.95±0.38 | 27.30±1.02 | 9.28±0.06 | 14.17±0.42 |

**4.** The column diagram of fluorescence intensity of Figure 7 is shown in Figure S12 (Red light). Red light indicates the fluorescence intensity of the dead cells.


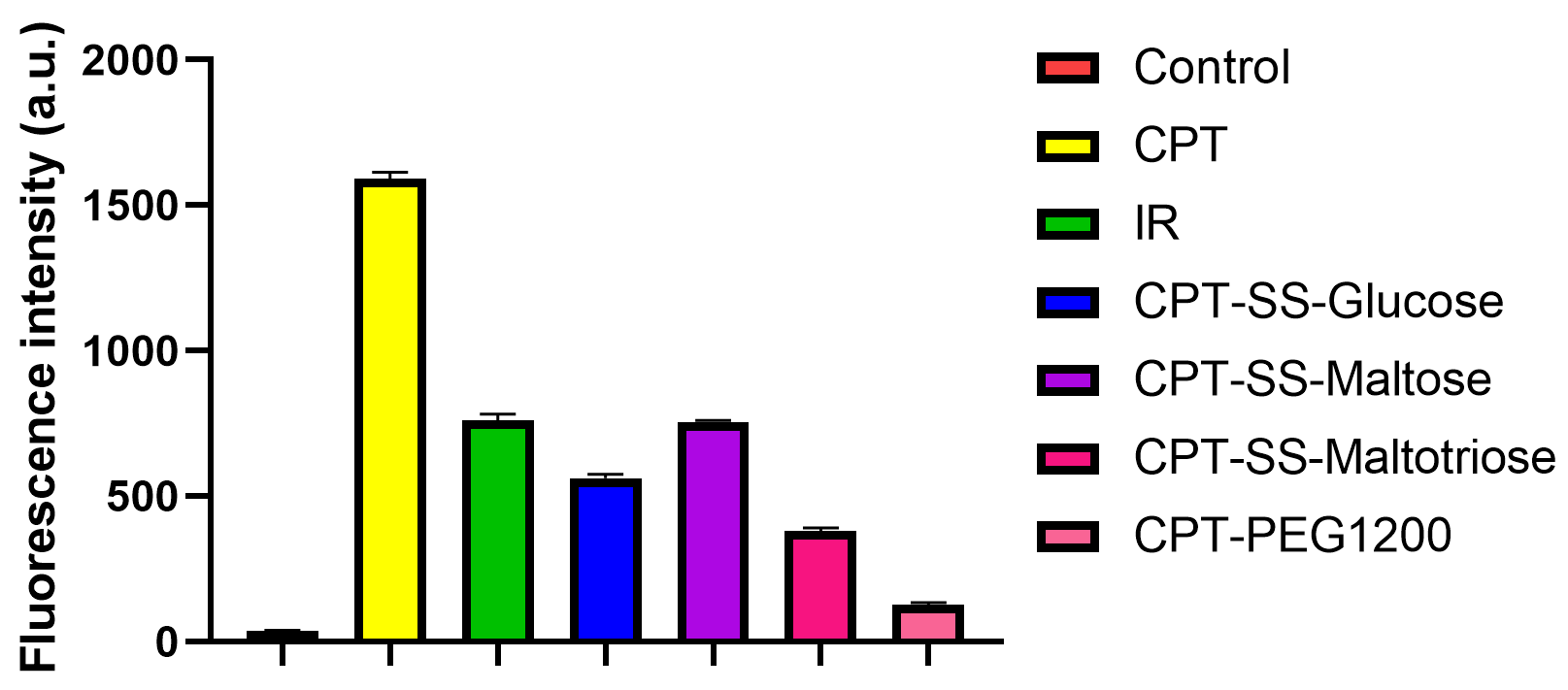


**Figure S12.** Fluorescence intensity of CPT, IR, and CPT-GL NSp.
